# Alzheimer's Disease in Ghanaian Medical Education: Investigating the Effect of Curriculum Changes on Student Perceptions and Attitudes

**DOI:** 10.1002/alz70860_096486

**Published:** 2025-12-23

**Authors:** Daniel Naawenkangua Abukuri

**Affiliations:** ^1^ University of Ghana, Accra, Greater Accra, Ghana

## Abstract

**Background:**

Alzheimer's disease is a progressive neurodegenerative disorder with significant public health implications. In Ghana, limited awareness among healthcare professionals often delays diagnosis and care. Medical students are crucial to addressing this gap, yet their training on AD remains inadequate. This study evaluated the impact of an educational program on medical students’ attitudes toward AD at the University of Ghana.

**Method:**

A quasi‐experimental design with pre‐ and post‐tests was used. A total of 160 medical students participated in a two‐week intervention comprising lectures on AD, workshops on patient communication and caregiving, and case studies. Attitudes were assessed using the Attitudes Toward Alzheimer's Disease (ATAD) scale, and qualitative insights were gathered through focus group discussions. Quantitative data were analyzed with paired t‐tests, and qualitative data were thematically analyzed

**Result:**

The intervention led to a significant improvement in medical students’ attitudes toward Alzheimer's Disease (AD). The mean ATAD (Attitudes Toward Alzheimer's Disease) scores increased from 45.2 ± 6.8 pre‐intervention to 72.4 ± 5.3 post‐intervention, demonstrating a statistically significant change (*p* < 0.001). This increase in scores reflects a notable shift in students' perceptions of AD. Additionally, 85% of participants reported feeling more confident in managing AD cases following the intervention. Qualitative feedback indicated that 90% of participants found the interactive learning formats, including workshops and case studies, to be highly beneficial. These methods were particularly praised for fostering greater empathy, improving understanding of the challenges associated with AD, and enhancing the overall learning experience. Furthermore, 80% of students expressed a stronger sense of responsibility and readiness to address dementia‐related concerns in clinical settings.

**Conclusion:**

The educational program effectively improved medical students’ attitudes and preparedness for managing Alzheimer's disease. Integrating similar training into medical curricula could enhance care for AD patients in Ghana, reduce stigma, and improve healthcare outcomes. Further research should examine the long‐term effects and scalability of these interventions.

